# Brain microstructural changes and cognitive correlates in patients with pure obsessive compulsive disorder

**DOI:** 10.1002/brb3.212

**Published:** 2014-01-27

**Authors:** Gianfranco Spalletta, Fabrizio Piras, Sabrina Fagioli, Carlo Caltagirone, Federica Piras

**Affiliations:** 1 Department of Clinical and Behavioral Neurology, Neuropsychiatry Laboratory, IRCCS Santa Lucia FoundationVia Ardeatina 306, 00179, Rome, Italy; 2 Department of Neuroscience, Tor Vergata University of RomeRome, Italy

**Keywords:** DTI, neuroimaging, neuropsychology, obsessive compulsive disorder, VBM

## Abstract

**Object:**

The aim of this study was to investigate macrostructural and microstructural brain changes in patients with pure obsessive compulsive disorder (OCD) and to examine the relationship between brain structure and neuropsychological deficits.

**Method:**

20 patients with OCD underwent a comprehensive neuropsychological battery. A combined voxel-based morphometry (VBM) and diffusion tensor imaging (DTI) analysis was used to capture gray matter (GM) and white matter changes in OCD patients as compared to pair-matched healthy volunteers. Multiple regression designs explored the relationship between cognition and neuroimaging parameters.

**Results:**

OCD patients had increased mean diffusivity (MD) in GM nodes of the orbitofronto-striatal loop (left dorsal anterior cingulate [*Z* = 3.67, *P* < 0.001] left insula [*Z* = 3.35 *P* < 0.001] left thalamus [*Z* = 3.59, *P* < 0.001] left parahippocampal gyrus [*Z* = 3.77 *P* < 0.001]) and in lateral frontal and posterior associative cortices (right frontal operculum [*Z* = 3.42 *P* < 0.001], right temporal lobe [*Z* = 3.79 *P* < 0.001] left parietal lobe [*Z* = 3.91 *P* < 0.001]). Decreased fractional anisotropy (FA) was detected in intrahemispheric (left superior longitudinal fasciculus [*Z* = 4.07 *P* < 0.001]) and interhemispheric (body of corpus callosum [CC, *Z* = 4.42 *P* < 0.001]) bundles. Concurrently, the semantic fluency score, a measure of executive control processes, significantly predicted OCD diagnosis (Odds Ratio = 1.37; 95% Confidence Intervals = 1.09–1.73; *P* = 0.0058), while variation in performance was correlated with increased MD in left temporal (*Z* = 4.25 *P* < 0.001) and bilateral parietal regions (left *Z* = 3.94, right *Z* = 4.19 *P* < 0.001), and decreased FA in the right posterior corona radiata (*Z* = 4.07 *P* < 0.001) and the left corticospinal tract (*Z* = 3.95 *P* < 0.001).

**Conclusions:**

The reported deficit in executive processes and the underlying microstructural alterations may qualify as behavioral and biological markers of OCD.

## Introduction

Current conceptualization of obsessive compulsive disorder (OCD) suggests that neurobiological abnormalities are crucial factors mediating the etiology and phenomenology of the illness. The most widely accepted model of OCD proposes that abnormalities of corticostriatal circuits, involving the orbitofrontal cortex (OFC), anterior cingulate cortex (ACC), thalamus and striatum play an important role in its pathophysiology (Graybiel and Rauch 2000; Saxena and Rauch 2000; Menzies et al. 2008a; Harrison et al. 2009). Such biological model has been partially validated by direct and indirect investigations of the possible circuits involved in the pathogenesis of the disorder. Specifically, functional neuroimaging studies, providing in vivo evidence of brain abnormalities in OCD patients, showed hyperactivity in orbitofronto-striatal circuits both in a resting state (Baxter et al. 1988) and during periods of provoked OCD symptoms (Rauch et al. 1994). Concurrently, a number of voxel-based morphometry (VBM) investigations have shown increased gray matter (GM) volume in the OFC and other cerebral structures belonging to the orbitofronto-striatal loop of OCD patients (Scarone et al. 1992; Kim et al. 2001; Valente et al. 2005; Christian et al. 2008), although findings have been conflicting with reports of reduced brain volume in the same regions (Szeszko et al. 1999; Pujol et al. 2004; Christian et al. 2008; Menzies et al. 2008a; Lázaro et al. 2009) or no morphometric differences between OCD patients and healthy control (HC) subjects (Jenike et al. 1996; Bartha et al. 1998).

In recent years, whole-brain-based VBM analyses have provided evidence that abnormalities in brain of OCD patients are not limited exclusively to the affective orbitofrontal loop, but extend to the dorsolateral prefrontostriatal loop (Piras et al. 2013a) and reciprocally connected temporo-parieto-occipital associative areas (Valente et al. 2005; Szeszko et al. 2008; Yoo et al. 2008; Togao et al. 2010). Complementary studies have also suggested macrostructural and microstructural brain abnormalities in OCD spreading beyond the GM nodes of the implicated corticostriatal corticothalamic pathways and involving white matter (WM) tracts that physically and functionally connects the nodes of these circuits. Moreover, brain WM changes in OCD patients have been found not only near regions more traditionally associated with the disorder (den Braber et al. 2011), but also in areas outside the orbitofronto-striatal circuit such as the dorsolateral prefrontal cortex (den Braber et al. 2011), and temporal, parietal, occipital regions (Szeszko et al. 2005; Kopřivová et al. 2009; Nakamae et al. 2011; Piras et al. 2013b).

Likewise, from a cognitive perspective, there has been sparse and inconsistent documentation of impairments in OCD patients on tasks classically defined as ‘orbitofrontal-dependent’, yet paradoxically other cognitive processes, not regarded to rely so heavily on orbitofrontal function, are frequently impaired in patients (Menzies et al. 2008a). In addition, studies examining the neuropsychological correlates of GM structural alterations showed that the most prevailing cognitive deficits in OCD (i.e., slower processing speed, response inhibition deficits and visuospatial memory impairments) were associated both with brain volumetric changes in the OFC (Jenike et al. 1996; Grachev et al. 1998; Choi et al. 2004; Menzies et al. 2007) but also with changes in regions outside the orbitofrontal loop such as the parietal cortex, the cerebellum (Menzies et al. 2007) and the dorsolateral prefrontal GM (Christian et al. 2008). Taken together, the reported evidence of neurobiological abnormalities in OCD suggests that the orbitofronto-striatal model may not be sufficient to fully explain the brain basis of the disorder (Piras et al. 2013a). From a neurobiological perspective, the “multiple-region pathogenesis” of OCD could be viewed as supportive of the notion that OCD emerges from disordered macro and microstructure as well as function, of large scale neural systems (Menzies et al. 2008a). The concurrent observation of WM abnormalities in OCD patients may also suggest that the disorder could be hypothetically underpinned by disconnectivity of such neurocognitive networks (Menzies et al. 2008b; Garibotto et al. 2010; Piras et al. 2013b). Then again, from a cognitive point of view, abnormalities across several brain regions and dysfunctionality at the system level could account for the reported incongruence between cognitive findings and the orbitofronto-striatal brain model of OCD. On the other hand, as heterogeneity in OCD neuroimaging originates from multiple differences across studies, first among many the clinical characteristics of the patient sample, it might be the case that the emerging picture of widespread structural brain changes in OCD is accounted for by the complex phenomenology of the disorder. Thus, in studying the association between brain and cognitive dysfunction in OCD the indices of cerebral abnormalities (e.g., volume reduction or diffusivity increase) have been used separately in different studies. As a consequence, a coherent picture of the neural basis of cognitive deficits in OCD is still lacking.

This study is aimed at filling this gap by analyzing brain macro and microstructural alterations and cognitive performances in a sample of OCD patients. In particular, the goal of the investigation is threefold: (1) by adopting a multivariate approach, we searched, among several neuropsychological variables, those cognitive impairments that could significantly predict OCD diagnosis; (2) by using a combined volumetry (VBM) and diffusion tensor imaging (DTI) method we investigated macrostructural-volumetric and microstructural-diffusivity brain changes in GM and WM that could differentiate a sample of “pure” OCD patients from matched HC subjects, and (3) in order to clarify the direction of causality between brain structure and neuropsychological deficits, we investigated the macrostructural and microstructural correlates of the OCD cognitive profile.

## Materials and Methods

### Subjects

Thirty-three patients meeting DSM-IV-TR criteria for OCD (American Psychiatric Association 2000) were consecutively approached from the IRCCS Santa Lucia Foundation in Rome. Diagnosis of OCD was made by a senior research psychiatrist (G. S.) who was also the clinician in charge of the patients' treatment, acquainted with their clinical history. All diagnoses were confirmed using the Structured Clinical Interview for DSM-IV-TR (SCID)-Patient Edition (First et al. 2002a).

Clinical history was collected from patients' physician or psychiatrist and clinical charts and eventually supplemented by interviewing the patients and their relatives. Symptom severity was assessed by a senior psychologist, PhD level, using the 10-item clinician-rated Yale-Brown Obsessive Compulsive Scale (Y-BOCS) (Goodman et al. 1989). Patients were also screened for the presence of general anxiety and depressive symptoms through the administration of the Hamilton Anxiety Rating Scale (HAM-A, Hamilton 1959) and the Hamilton Depression Rating Scale (HAM-D, Hamilton 1960).

Exclusion criteria included: (1) comorbid psychiatric disorders according to DSM-IV-TR criteria, (2) a history of psychoactive substance dependence or abuse during lifetime, (3) a history of neurologic illness or brain injury, (4) major medical illnesses, that is, diabetes not stabilized, obstructive pulmonary disease or asthma, hematological/oncological disorders, B12 or folate deficiency as evidenced by blood concentrations below the lower normal limit, pernicious anemia, clinically significant and unstable active gastrointestinal, renal, hepatic, endocrine or cardiovascular system disease, newly treated hypothyroidism, (5) the presence of any brain pathology as instantiated by standard magnetic resonance imaging (MRI) exams (including T1, T2 and FLAIR protocols). In particular, the presence, severity and location of vascular lesions were rated according to a protocol designed for the Rotterdam Scan Study (de Leeuw et al. 2001). They are considered present in cases of hyperintense lesions on both proton-density and T2-weighted and were rated semiquantitatively as 0 (none), 1 (pencil- thin lining), 2 (smooth halo) or 3 (large confluent) for three separate regions; adjacent to frontal horns (frontal caps), adjacent to the wall of the lateral ventricles (bands) and adjacent to the occipital horns (occipital caps). The total vascular lesion load was calculated by adding the region-specific scores (range, 0–9). In this study, only patients rated 0 were included, (6) global cognitive deterioration according to a Mini-Mental State Examination (MMSE) (Folstein et al. 1975) score lower than 26, (7) premorbid IQ below the normal range according to TIB (Test Intelligenza Breve, Italian analog of the National Adult Reading Test – NART – Nelson 1982) cutoff score of 93.1 (Sartori et al. 1997), and (8) Dementia diagnosis according with DSM-IV-TR criteria (see cognitive assessment for neuropsychological measurements).

By applying exclusion criteria, the OCD patients included in the final sample were reduced to a total of 20 (four could not undergo MRI scan because of claustrophobia, two were excluded due to artifacts in MRI images, three had evidence of cerebrovascular lesions and four had psychiatric comorbidity). Sociodemographic and clinical characteristics of the sample are shown in Table 1.

**Table 1:** Sociodemographic and clinical characteristics of 20 patients with OCD and 20 HC subjects

Variables	Patients (*n* = 20) Mean (SD)	Controls (*n* = 20) Mean (SD)	*t*	*df*	*P*
Age	33.10 (8.85)	35.20 (9.38)	−0.72	38	0.40
Educational attainment	12.65 (3.6)	13.55 (3.51)	−0.84	38	0.40
Duration of illness	13.52 (10.84)	–	–	–	–
Y-BOCS symptom severity score	24.78 (6.68)	–	–	–	–
Hamilton Anxiety Scale score (HAM-A)	17.5 (8.8)	2.7 (2.9)	7.17	38	<0.001
Hamilton Depression Rating Scale (HAM-D)	12.1 (6)	2.3 (2.3)	6.5	38	<0.001
Olanzapine equivalents (mg/d)	9.25 (5.19)	–	–	–	–
Venlafaxine equivalents (mg/d)	244.93 (120.18)	–	–	*–*	*–*
Diazepam equivalents (mg/d)	9.73 (9.81)	–	–	–	–
	*N* (%)	*N* (%)	χ^2^	*df*	*P*
Gender (male)	12 (60)	12 (60)	0.00	1	0.99
On antipsychotics	12 (60)	–	–	–	–
Combined treatment (antipsychotics and antidepressants)	11 (55)	–	–	–	–
On anxiolytics	12 (60)	–	–	–	–

It is important to highlight that the low rate (12%) of psychiatric comorbidity in our OCD sample (before exclusion criteria were applied), was determined by the implementation of a preselection strategy based on a general clinical interview conducted by experienced clinicians, such that no patient with evident psychiatric comorbidities was considered for inclusion. At the time of testing, 60% of OCD patients (*n* = 12) were taking oral doses of atypical or classical antipsychotic drugs such as quetiapine (two patients), aloperidol (two patients), olanzapine (three patients), ziprasidone (two patients), phenotiazine (one patient), and risperidone (two patients). Antipsychotic dosages were converted to equivalents of olanzapine. A total of 11 patients were on combination of antidepressants and atypical (four patients) or typical (seven patients) antipsychotics. Antidepressant dosages were converted to equivalents of venlafaxine. Sixty percent of patients (12) were receiving stable dosages of benzodiazepines, which were converted to equivalents of diazepam. Pharmacological treatment is shown in Table 1.

Twenty HC subjects, one to one pair-matched by age, sex and educational level (see Table 1), were recruited from the same geographical area. All the HC subjects were carefully screened for a current or past diagnosis of any DSM-IV-TR Axis I or II disorder using the SCID-I nonpatient edition (First et al. 2002b) and SCID-II (First et al. 1997). The presence of major medical illnesses was an exclusion criterion as well as the other above-mentioned exclusion criteria for OCD patients.

All participants were right-handed. They gave written informed consent to participate after the procedures had been fully explained. The study was approved and carried out in accordance with the guidelines of the IRCCS Santa Lucia Foundation Ethics Committee.

### Cognitive assessment

After having being screened for global cognitive impairment using the Mini-Mental State Examination test (Folstein et al. 1975), all study subjects underwent a comprehensive neuropsychological battery performed by a trained neuropsychologist. The Trail-Making Test-parts A and B (TMT-A and TMT-B) (Reitan 1992) were administered to evaluate speed of information processing (TMT-A) and set-switching ability as a measure of cognitive flexibility and executive functioning (TMT-B). The Controlled Word Fluency Test (WFT) from the Mental Deterioration Battery (MDB), a validated instrument to detect early cognitive decline (Carlesimo et al. 1996), and the Semantic Fluency Test (SFT) (Lucas et al. 1998) were used to assess the phonological and semantic processes central to speech production and the executive processes implied in word search and switching between subcategories. The Rey's 15 word Immediate and Delayed Recall test from the MDB (Carlesimo et al. 1996) measured subjects' declarative verbal memory, while the Rey–Osterrieth Complex Figure Test immediate copy (ROCFT) (Osterrieth 1944) evaluated visuoconstructive abilities.

### Image acquisition and processing

Participants underwent the same imaging protocol, which included standard clinical sequences (FLAIR, DP-T2-weighted), whole-brain 3D high-resolution T1-weighted and diffusion-weighted scanning using a 3T Allegra MR imager (Siemens, Erlangen, Germany). Volumetric whole-brain T1-weighted images were obtained using a modified driven equilibrium Fourier transform (MDEFT) sequence (TE/TR = 2.4/7.92 msec, flip angle 15º, voxel size 1 × 1 × 1 mm^3^). Diffusion-weighted volumes were acquired using echo-planar imaging (TE/TR = 89/8500 msec, bandwidth = 2126 Hz/vx; matrix size 128 × 128; 80 axial slices, voxel size 1.8 × 1.8 × 1.8 mm^3^) with 30 isotropically distributed orientations for the diffusion-sensitizing gradients at a b value of 1000 sec mm^2^ and 6 b = 0 images. Scanning was repeated three times to increase the signal-to-noise ratio.

High-resolution T1-weighted and DTI images were processed separately to obtain indices of brain macro and microstructural alteration.

First, T1-weighted images were processed and examined using the SPM8 software (Wellcome Department of Imaging Neuroscience Group, London, UK; http://www.fil.ion.ucl.ac.uk/spm), specifically the VBM8 toolbox (http://dbm.neuro.uni-jena.de/vbm.html), running in Matlab 2007b (MathWorks, Natick, MA). The toolbox extends the unified segmentation model (Ashburner and Friston 2005) consisting of MRI field intensity inhomogeneity correction, spatial normalization and tissue segmentation at several preprocessing steps in order to further improve the quality of data preprocessing. Initially, in order to increase the signal-to-noise ratio in the data, the optimized blockwise nonlocal-means filter proposed by Coupé et al. (2006) was applied to the MRI scans using the Rician noise adaption (Wiest-Daessl et al. 2008). Then, an adaptive maximum a posteriori segmentation approach extended by partial volume estimation (Manjón et al. 2008) was employed to separate the MRI scans into GM, WM and cerebrospinal fluid (CSF). The segmentation step was finished by applying a spatial constraint to the segmented tissue probability maps based on a hidden Markow Random Field model (Cuadra et al. 2005) in order to remove isolated voxels which were unlikely to be a member of a certain tissue class and to close holes in clusters of connected voxels of a certain class, resulting in a higher signal-to-noise ratio of the final tissue probability maps. Then, the iterative high-dimensional normalization approach provided by the Diffeomorphic Anatomical Registration Through Exponentiated Lie Algebra (DARTEL) (Ashburner 2007; Bergouignan et al. 2009; Klein et al. 2009) toolbox was applied to the segmented tissue maps in order to register them to the stereotactic space of the Montreal Neurological Institute (MNI). The tissue deformations were used to modulate the participants' GM and WM tissue maps in order to compare volumetric differences across groups. Voxel values of the resulting normalized and modulated GM and WM segments indicate the probability (between 0 and 1) that a specific voxel belongs to the relative tissue. Finally, the modulated and normalized GM and WM segments were written with an isotropic voxel resolution of 1.5 mm^3^ and smoothed with a 6 mm full with half maximum (FWHM) Gaussian kernel, thus obeying the ‘rule of thumb’ that the FWHM should be at least twice the voxel dimension in order to ensure a Gaussian distribution of the residuals of the General Linear Model (Ashburner, personal communication via SPM mailing list 2004; Moraschi et al. 2010).

Subsequently, DTI images were processed using FSL 4.1 (http://www.fmrib.ox.ac.uk/fsl/). Image distortions induced by eddy currents and head motion in the DTI data were corrected by applying a 3D full-affine (mutual information cost function) alignment of each image to the mean no diffusion weighting (b0) image. After these corrections, DTI data were averaged and concatenated into 31 (1 b0 + 30 b1000) volumes. A diffusion tensor model was fit at each voxel, generating FA and mean diffusivity (MD) maps.

FA maps were processed using Tract-Based Spatial Statistics (TBSS) (Smith et al. 2006), part of FSL. All subjects' FA data were aligned into a common space using the nonlinear registration tool FNIRT (FMRIB's Non-linear Image Registration Tool, FSL), which uses a b-spline representation of the registration warp field. Next, the mean FA image was created and thinned to create a mean FA skeleton, which represents the centers of all tracts common to the group. Each subject's aligned FA data was then projected onto this skeleton and the resulting data fed into voxelwise cross-subject statistics.

On the other hand, MD maps were first aligned to the Montreal Neurological Institute (MNI) template using the FNIRT tool, and subsequently smoothed with a Gaussian kernel with FWHM of 6 mm.

### Statistical analysis

#### Behavioral

Demographic data were compared using Student *t*-test for age and educational level and chi-square test for gender.

In order to identify neuropsychological variables significantly differentiating OCD cases and HC, a multivariate logistic regression modeling was used to estimate the odds of independent variables that differed significantly, at a *P* < 0.05 level, between HC and OCD (with diagnosis as dependent variable). The multivariate model was chosen in order to minimize the likelihood of type-I (false-positive results) errors. Preselection of independent variables to include in the multivariate logistic regression model was done by using Student *t*-test analysis in order to determine the significance of differences between OCD patients and HC. In the multivariate logistic model, only individual variables with *P* < 0.05 in the preselection analyses were included. All tests were two-tailed, and the level of statistical significance was defined as *α* < 0.05. Because of multicollinearity between neuropsychological variables, which impacts conclusions about the significance of effects and model applicability in regression models, we checked, separately for the two groups, the tolerance value of each viable predictor, that is, the proportion of variation in each predictor independent from the correlation between regressors (Berk 1977). The tolerance value was computed as:


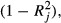


where *R*^2^_*j*_ is the coefficient of determination obtained by modeling the *j*^*th*^ regressor (each neuropsychological test score where a significant difference between OCD and HC was observed) as a linear function of the remaining independent variables. The cutoff value was set such that the variability in a predictor not related to other variables in the model was at least larger than 30%.

#### Neuroimaging

In order to avoid possible edge effects between different tissue types, the VBM analyses of GM and WM volumes were carried out excluding all voxels with a probability of belonging to the relative tissue less than 20% (absolute threshold masking). Further, statistical analyses of MD maps were restricted to cortical and deep GM structure using an inclusive mask obtained by averaging subjects' GM segments and excluding all voxels with a probability of belonging to GM less than 30%. Finally, statistical analyses on FA maps were restricted to voxels in the WM skeleton.

Differences in four neuroimaging parameters (i.e. GM and WM volume, GM MD and WM tract FA) between HC and OCD subjects were tested at the voxel level by means of unpaired *t*-tests using SPM-8 within the framework of the General Linear Model.

The relationship between cognition and neuroimaging parameters in the two groups was assessed as follows: subjects' scores in the neuropsychological task resulting as the only significant predictor of diagnosis (i.e., SFT, see 2013 section) was entered as regressor in eight different multiple regression designs (i.e., two groups – OCD and HC subjects – and four imaging parameters – GM and WM volume, GM MD and WM tract FA), using age and years of formal education as covariates of no interest.

Finally, to obtain fine anatomical connectivity localization of statistical results on GM and WM, respectively, two different brain atlases were used: (1) the automated anatomical labeling (AAL) (Tzourio-Mazoyer et al. 2002), which includes all main gyri and sulci of the cerebral cortex and the subcortical and deep gray matter structures for a total of 90 anatomical volumes of interest, and (2) the ICBM-DTI-81 white matter labels atlas (Mori et al. 2008), which includes 50 WM tract labels created by hand segmentation of a standard-space average of diffusion MRI tensor maps from 81 subjects. Statistical threshold was set at *P* < 0.001, which is a relatively lenient threshold and a good trade-off between the control of false positive and reliability (Thirion et al. 2007).

## Results

### Sociodemographic and neuropsychological variables

As expected from the matching procedure, the two groups did not significantly differ for age, educational attainment and gender (see Table 1).

Exploratory individual analyses revealed that the two diagnostic groups significantly differed relative to the Rey's 15 word Immediate and Delayed Recall score, the TMT-B score and the SFT score. Specifically, OCD patients scored lower than HC in the Rey's 15 word Immediate and Delayed Recall and the SFT, while needed significantly more time than controls to complete the TMT-B task (see Table 2).

**Table 2:** Neuropsychological performance of 20 patients with OCD and 20 HC subjects

Variables	Patients (*n* = 20) Mean (SD)	Controls (*n* = 20) Mean (SD)	*t*	df	*P*
MMSE	28.85 (1.08)	29.4 (0.68)	−1.91	38	0.06
TMT–A time (sec)	70.15 (30.07)	54.3 (12.29)	1.95	38	0.057
TMT–B time (sec)	171.40 (83.46)	110.95 (37.38)	**2.80**	**38**	**0.007**
WFT	29.38 (10.44)	34.95 (10.75)	−1.77	38	0.08
SFT	14.55 (4.71)	23.40 (4.42)	**−6.11**	**38**	**<0.001**
Rey's 15 words immediate recall	34.41 (10.72)	47.01 (8.26)	**−3.81**	**38**	**0.0005**
Rey's 15 words delay recall	6.31 (2.91)	9.54 (2.33)	**−3.65**	**38**	**0.008**
ROCFT immediate copy	29.23 (6.27)	31.57 (2.05)	−1.64	38	0.10

Significant values at *P* < 0.05 are highlighted in bold. MMSE, mini-mental state examination; TMT, trail-making test; WFT, controlled word fluency test; SFT, Semantic fluency test; ROCFT, Rey–Osterrieth complex figure test.

However, the collinearity among test variables was high in both groups as, for example, less than 30% of the variance associated with the Rey's 15 word Immediate Recall score in the HC group was independent of other predictors (see Table 3). The latter variable was therefore excluded from the subsequent multivariate logistic regression, as the inclusion of both the Rey's 15 word Immediate and Delayed Recall score would not have added more information to the model than the inclusion of just one of them.

**Table 3:** Tolerance value for the neuropsychological variables where a significant difference between OCD and HC was observed

Variables	Patients (*n* = 20) Independent variable (%)	Controls (*n* = 20) Independent variable (%)
TMT–B time (sec)	74	90
SFT	85	94
Rey's 15 words immediate recall	30	29
Rey's 15 words delay recall	28	31

The value is expressed as the percentage of variation in each test score independent from the correlation among neuropsychological variables. TMT, Trail-Making Test; SFT, Semantic fluency test.

The overall model including the Rey's 15 word Delayed Recall score, the TMT-B score and the SFT score as predictor variables and the diagnostic group as dependent variable was significant (likelihood ratio: *χ*^2^ = 27.76; df = 3; *P* < 0.001) and explained 50% of the total variance (adjusted *R*^2^). Specifically, the SFT score was the only significant predictor of diagnosis (Odds Ratio [OR] = 1.37; 95% Confidence Intervals (CI) = 1.09–1.73; *P* = 0.0058] so that the odds of belonging to the OCD group increased about 1.4 times for each word omitted in the SFT. The overall prediction accuracy of the model was 87.50%, while 90% of OCD patients could be accurately classified on the basis of the SFT score.

### Neuroimaging

#### Cortical/deep structures GM analysis and neuropsychological correlates

Results of macrostructural-VBM analysis revealed no GM volumetric differences between OCD patients and HC subjects. Therefore, no correlation between GM volumetric measures and cognitive performance was examined.

**Figure 1:**
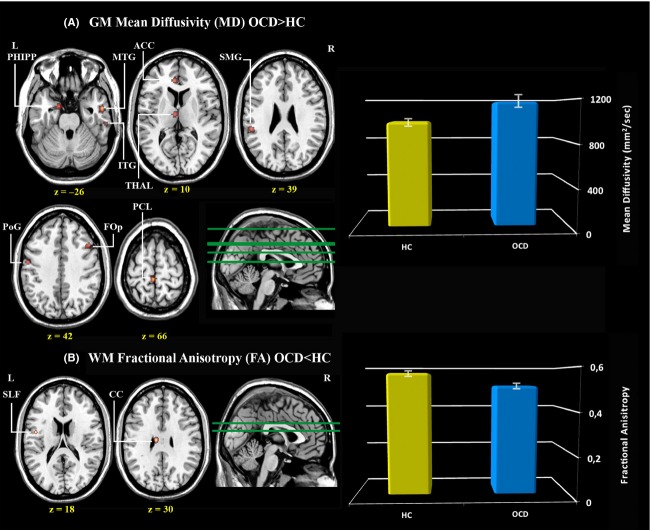
Brain gray matter and white matter microstructure of 20 patients with OCD compared to 20 HC subjects. Brain regions where significant differences between patients with obsessive compulsive disorder and healthy controls were found in microstructural-diffusivity measures of gray matter (A) and white matter (B) integrity. ACC, anterior cingulate cortex; CC, corpus callosum; FOp, frontal operculum; GM, gray matter; HC, healthy controls; ITG, inferior temporal gyrus; L, left; MTG, medial temporal gyrus; OCD, obsessive compulsive disorder; PCL, precentral gyrus; PHIPP, parahippocampus; PoG, postcentral gyrus; R, right; SLF, superior longitudinal fasciculus; SMG, supramarginal gyrus; THAL, thalamus; WM, white matter.

At the microstructural-DTI level, the *t*-test comparison between OCD patients and HC subjects on the GM MD (see Table 4A) showed increased MD values in OCD in the left paracentral lobule, the left anterior cingulum, the left supramarginal and postcentral gyri within the parietal lobe, the right temporal medium and inferior gyri and the left parahippocampal gyrus. Areas of increased MD were also found in the left thalamus (three contiguous regions), in a small cluster in the left insula and in the right frontal operculum. No areas of decreased MD were found in the OCD sample in comparison with HC subjects (Fig. 1, panel A).

**Table 4:** Brain microstructural changes in 20 OCD patients in comparison to 20 HC subjects

	Labels for peaks	Extent	Equivalents *Z*	x,y,z (mm)^2^
*(A) GM mean diffusivity*—*t*-*test comparison (OCD>HC)*^1^				
Anatomical region				
Frontal lobe	L paracentral lobule	20	4.28	−4, −32, 66
Cingulum	L anterior cingulum	54	3.67	−6, 32, −6
Parietal lobe	L supramarginal gyrus	13	3.91	−52, −34, 26
	L postcentral gyrus	13	3.57	−56, −6, 42
Temporal lobe	R temporal middle gyrus	23	3.79	54, −4, −26
	R temporal inferior gyrus	16	3.44	60, −26, −30
	L parahippocampal gyrus	58	3.77	−16, 0, −24
Thalamus	L thalamus	46	3.59	−2, −9, 4
		20	3.45	−4, −20, −6
		19	3.35	−6, −14, 8
Insula	L insula	14	3.35	−32, −20, 18
Frontal lobe	R frontal operculum	16	3.42	46, 18, 40
*(B) WM fractional anisotropy*—*t*-*test comparison (OCD*<*HC)*^1^				
Anatomical region				
Corpus callosum	L body of corpus callosum	13	4.42	−10, −15, 30
Frontal lobe	L superior longitudinal fasciculus	13	4.07	−47, −2, 22

(A) Voxel-based gray matter mean diffusivity differences between OCD patients and HC subjects. (B) Tract-based spatial statistic (TBSS) analysis of white matter fractional anisotropy differences between OCD patients and HC subjects. R, Right; L, Left.

1 At *P* < 0.001 uncorrected.

2 Coordinates are in Montreal Neurological Institute Space.

**Figure 2:**
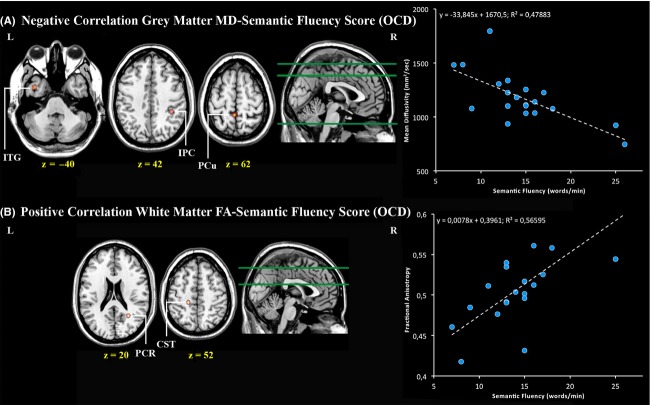
Neuropsychological microstructural correlates of obsessive compulsive disorder. Areas where significant correlations between microstructural-diffusivity measures of gray matter (A) and white matter (B) integrity and performance in a semantic fluency task were found limited to the obsessive compulsive population. The structure–function relationship is depicted in the pertaining graphs on the picture right side. CST, corticospinal tract; FA, fractional anisotropy; IPC, inferior parietal cortex; ITG, inferior temporal gyrus; L, left; MD, mean diffusivity; OCD, obsessive compulsive disorder; PCR, posterior corona radiate; PCu, precuneus; R, right.

In order to determine whether there was a relationship between the neuropsychological variable differentiating OCD cases and HC subjects and GM microscopic tissue structure, correlations between GM MD values and the SFT score were examined on a voxelwise basis in the two samples (see Table 5A). For the OCD patients group, a significant negative correlation was detected in the left inferior temporal gyrus, the left precuneus and the right inferior parietal gyrus, so that the semantic fluency score decreased as MD values increased in the reported areas (Fig. 2, panel A). Of note, the observed correlation was detected in areas different from those emerged as pathogenic for OCD in the unpaired *t*-test comparing GM MD values in the two groups. No significant correlation between the SFT score and GM microscopic tissue structure was observed in HCs.

**Table 5:** Brain microstructural correlates of semantic fluency test performances in OCD patients

Anatomical region	Labels for peaks	Extent	Equivalents *Z*	x,y,z (mm) 2
(A) Mean diffusivity—negative correlation MD-semantic fluency score (OCD)^1^				
Temporal lobe	L temporal inferior	21	4.25	−40, 4, −40
Parietal lobe	L precuneus	28	3.94	−2, −42, 62
	R parietal inferior	12	4.19	39, −39, 42
(B) WM fractional anisotropy—positive correlation FA-semantic fluency score (OCD)^1^				
Corona radiata	R posterior corona radiata	15	4.07	29, −57, 20
Parietal lobe	L corticospinal tract	13	3.95	−17, −30, 52

(A) Areas of significant correlation between gray matter mean diffusivity (MD) values and the semantic fluency score in the OCD sample. (B) Areas of significant correlation between white matter (WM) fractional anisotropy (FA) and the semantic fluency score in the OCD sample. R, Right; L, Left.

1 At *P* < 0.001 uncorrected.

2 Coordinates are in Montreal Neurological Institute Space.

#### WM analysis and neuropsychological correlates

As no significant differences were observed in WM volume among the two groups, no correlation between volumetric measures and cognitive performance was examined.

In the TBSS analysis, the unpaired *t*-test among groups on FA values showed a significant reduction in the whole OCD group in three clusters. Specifically, lower FA values in the OCD sample were found in the body of corpus callosum (CC) and in the left superior longitudinal fasciculus (SLF). No areas of increased FA were found in the OCD sample in comparison with HC subjects (Fig. 1, panel B). MNI coordinates of the above-mentioned tracts are shown in Table 4B.

The correlation analysis between FA values and the SFT score (see Table 2003B) showed a significant positive correlation in the OCD sample in a cluster comprising the posterior corona radiata in the right hemisphere and the corticospinal tract in the left hemisphere (Fig. 2, panel B). Then again, the structure–function relationship was observed in tracts distinct from those where reduced FA values were detected in the OCD group. Finally, no significant correlation between WM microstructure and the SFT score was found in HC subjects.

## Discussion

Although current approaches to OCD suggest that neurobiological abnormalities mediate the expression of the cognitive impairments associated with the disorder, limited investigations have aimed at characterizing the neural substrates of these functional deficits. Moreover, few studies to date (e.g., Garibotto et al. 2010) explored the potential correlation between microstructural damage and altered cognition in OCD, mainly limiting their investigation to measures of WM integrity.

Here, we analyze the neuropsychological profile of pure OCD patients and demonstrate that reduced semantic fluency is a neurocognitive marker of the illness. From a neuroanatomical perspective, microstructural abnormalities in lateral frontal, parietal, and temporal cortices and altered integrity in intra and interhemispheric associative tracts differentiated OCD patients from HCs. On the other hand, the semantic fluency impairment correlated with microstructural tissue damage in areas distinct from those identified as pathogenic in our OCD sample, suggesting that cognitive disturbance in OCD emerges from microstructural alterations in regions not directly involved in the disorder pathophysiology. However, it is also possible that studies using different neuroimaging techniques, measuring cerebral perfusion, metabolism, or neurochemistry may characterize complementary aspects of OCD pathways and neurobiological mechanisms, thus integrating results from structural MRI investigations and eventually capturing the relationship between abnormal brain activity and cognitive impairment in OCD patients (e.g., Nakao et al. 2009).

### Neural correlates of neuropsychological variables differentiating OCD patients from HC subjects

Published studies of neurocognitive functioning in OCD have yet to reveal a reliable cognitive signature of the disorder. While deficits in motor response inhibition, attentional set-shifting and impairments in planning aspects of executive functioning have been largely acknowledged (Nielen and den Boer 2003; van den Heuvel et al. 2005; Chamberlain et al. 2007), neuropsychological studies have also produced inconsistent findings, possibly as a consequence of heterogeneity of OCD regarding comorbidity (Nakao et al. 2009). In addition, previous investigations suggested no deficits in verbal fluency in OCD patients (Head et al. 1989; Martin et al. 1993; Bannon et al. 2006), while others report otherwise (Christensen et al. 1992; Schmidtke et al. 1998; Jurado et al. 2001; Lacerda et al. 2003; Roh et al. 2005; see Kuelz et al. 2004 for a review). In line with the current literature posing specific cognitive deficits in OCD (Cavedini et al. 2010), we found selective impairments in verbal declarative memory and in executive functioning measures of cognitive flexibility (TMT part B, Kortte et al. 2002) and strategic response organization (SFT, Salthouse et al. 2003). Moreover, by adopting a multivariate approach, which takes into account the effects of all neuropsychological variables on the response of interest, we identified the semantic fluency score as a predictive factor of diagnosis, as 90% of OCD patients could be accurately classified on the basis of the SFT score. Although limited to the present sample, such finding would indicate that reduced semantic fluency is the most distinctive cognitive trait of OCD and that the cognitive processing abnormalities underlying the observed deficit might be etiological relevant factors in the disorder, though distinct from other assumed pathogenic factors such as brain circuitry anomalies.

Indeed, individual variation in the semantic fluency performance was correlated in the OCD sample, with diffusivity changes in three areas (the left inferior temporal gyrus, the left precuneus and the right inferior parietal gyrus) so that the semantic fluency score decreased as MD values increased, though no microstructural differences were observed in these regions between OCD and HC. Actually, the role of the left temporal lobe in semantic fluency is well-established by functional imaging studies showing that particularly the left inferior temporal cortex is more involved in word retrieval according to given categories (e.g.: Gourovitch et al. 2000; Grogan et al. 2009; Mummery et al. 1996; Heim et al. 2008). On the other hand, the association between the semantic fluency score and the microstructural integrity of the left precuneus is less expected, although it might be related to use of a visual search strategy. Indeed, even if the categorical fluency task is primarily a semantic auditory task, participant often report recourse to strategies more based on visual imagery. It is thus conceivable that owing to the microscopic alterations in temporal structures responsible for semantic memory, OCD patients did not recur to a semantic association strategy to unravel the task, but rather relied on a visual imagery search strategy, though the latter was somehow impeded by loss of normal microstructure in the left precuneus. A negative correlation between performance in the semantic fluency task and microstructural tissue integrity was also observed in the right parietal cortex, a region probably implicated in the control of switching across different stimuli (see Gurd et al. 2002). As semantic fluency requires not only the ability to cluster words within a given category but also the capacity to switch efficiently to a new subcategory, it is possible that the observed loss of microstructural tissue integrity in the parietal lobe might have hindered the ability to shift efficiently between subcategories, hence determining the observed reduced category fluency in our OCD sample. A positive correlation between the semantic fluency score and FA values was also found for the OCD group, in the right posterior corona radiata (CR) and the left corticospinal tract (part of the CR), so that the number of generated words diminished as FA values decreased. Microstructure integrity of the posterior corona radiata has been previously related to general processing speed and episodic memory retrieval (Bendlin et al. 2010), while reduced integrity in the right corticospinal tract predicted executive dysfunction in traumatic brain-injured patients (Kinnunen et al. 2011). Thus, microstructural damage of tracts connecting frontal lobes to more posterior brain regions might have disturbed the executive performance component of the semantic fluency task (Troyer et al. 1997; Beatty et al. 2000; Rosen et al. 2005) determining the observed reduced word generation in our OCD sample.

An important remark is that the reported structure–function relation was found in areas different from those emerged as pathogenic for the illness in our OCD sample. Even if at this time the role of general cognitive deficits in the etiology and persistence of OCD remains unknown, such result would suggest that brain abnormalities playing a crucial role in OCD etiology do not mediate the expression of the cognitive impairments associated to the illness. On the other hand, performance deficits are not necessarily just attributable to structural impairment in specific regions (Krishna et al. 2011) as cognitive functioning is most likely to recruit multiple neural networks and to emerge through interregional functional connectivity. Indeed, studies investigating brain dysfunction of OCD during the resting state, and thus examining neural mechanisms not specific to the task employed, confirmed altered functional connectivity (Stern et al. 2012) and abnormal spontaneous neuronal activity (Hou et al. 2012) not only in the affective circuit thought to be involved in OCD pathogenesis but also in a broader set of cortical regions, including the parietal cortex. As more posterior cortices are involved in various cognitive functions, the observed dysfunction in large-scale neuronal circuits may well account for both disease expression and impairments in cognition.

Alternatively, it is possible that the subtle microstructural alteration underlying the observed cognitive deficits could be an early feature of neurobiological abnormalities eventually leading to the subsequent emergence of OCD symptoms. As a matter of fact, studies investigating the nature of cognitive impairment in OCD demonstrated that the neurocognitive profile did not vary with the duration of the disease (Trivedi et al. 2008), while the neuropsychological performance was independent from the level of clinical improvement due to pharmacological treatment (Kim et al. 2002; Nielen and den Boer 2003). Such findings would indicate the relative stability over time of the brain dysfunction responsible for impaired cognition, suggesting that the neural system underlying cognitive deficits does not directly mediate obsessive-compulsive symptoms (Nielen and den Boer 2003).

### Brain abnormalities in OCD patients

A second result is that we did not find, in the studied OCD sample, any macrostructural-volumetric alteration of neither GM nor WM. Although the findings from volumetric imaging studies of OCD have been fairly inconsistent, with reports of either increases or decreases (Szeszko et al. 1999) in brain regions thought to be implicated in the pathophysiology of the disorder, our result is consonant with previous investigations that also failed to detect any macrostructural difference between groups of OCD patients and HC subjects (Jenike et al. 1996; O'sullivan et al. 1997; Rosenberg et al. 1997; Bartha et al. 1998; Riffkin et al. 2005). We already discussed the sources of discrepancy in volumetric studies of OCD, however, it is also possible that abnormalities at the microstructural level, as investigated using DTI, could play a role in the neuropathology of the disorder (Szeszko et al. 2005). Indeed, we did find microstructural diffusivity changes in our OCD patients, with increased MD in several cortical regions (left dorsal ACC, insula, thalamus and parahippocampal gyrus, right frontal operculum and temporal lobe, left parietal lobe) and reduction in FA values (a putative measure of fibre density, axonal diameter and myelination) in two WM tracts (the left SLF and the body of CC). As both diffusion indices are used to interrogate pathological changes in cerebral tissue and probe the integrity of WM fibre tracts (Basser and Jones 2002), we can assume that altered architecture in specific cortical areas and WM tracts may be responsible for OCD pathophysiology.

Provided that there are no previous DTI studies examining brain cortical MD in OCD patients, our results cannot be compared with other investigations, although volumetric neuroimaging studies may supply some insight into the role of the aforementioned areas in OCD pathogenesis. Actually, compelling evidence suggests that abnormalities in orbitofrontal, cingulate, thalamic, and temporolimbic regions play a central role in the pathophysiology of OCD (Piras et al. 2013a). The pattern of brain alterations in OCD patients is characterized by reduced volume in the cingulate gyrus, and increased volume in the putamen, striatum, thalamus, and temporolimbic regions, suggesting that volume reduction in the cortical source of the orbitofronto-striatal loop, and relative expansion of tissue at the deep GM nuclei and limbic level, may have a primary role in OCD (Pujol et al. 2004; Piras et al. 2013a). Also the insular cortex, a region directly linked to the ventral part of the striatum and probably functionally related to the frontostriatal system, has been implicated in the pathogenesis of OCD by VBM studies showing either increased GM volume in the right and left insula (Valente et al. 2005) or volume reduction in the same regions (Pujol et al. 2004; Yoo et al. 2008).

On the other hand, morphometric alterations in OCD seem to involve a more broadly distributed set of cortical areas within frontal, temporal, and parietal lobes (Menzies et al. 2008a; Piras et al. 2013a). For example, emerging work suggests that a region comprising the frontal operculum and the anterior insula, is a key structure in an extended network (comprising also the medial frontal cortex, the dorsal ACC and the OFC) for evaluating the emotional/motivational salience of errors (Ullsperger et al. 2010; Stern et al. 2011). The fact that we observed microstructural changes in several nodes of such error-detection system (left dorsal ACC, left insula and right frontal operculum) suggests that abnormalities within this brain network may play an important role in the pathogenesis of OCD, where pathological levels of importance are attributed to simple behavioral (or perceived) errors.

Interestingly, we also found evidence of microstructural diffusivity changes in posterior brain regions such as the postcentral and supramarginal gyri, and the temporal medium and inferior gyri.

Regarding the parietal lobe, our finding of microstructural alterations in the left postcentral and supramarginal gyri are consistent with the growing body of evidence implicating dysfunctions in these structures in the pathophysiology of OCD. Direct confirmation comes from reports of hypometabolism in the lateral parietal cortex in OCD patients (Lucey et al. 1995; Nordahl et al. 1998; Kwon et al. 2003) and from VBM studies showing decreased GM volume in the parietal lobe (Valente et al. 2005; Carmona et al. 2007; Yoo et al. 2008; Kopřivová et al. 2009; Lázaro et al. 2009, 2011). Moreover, given that visuospatial abilities, nonverbal memory and attentional shifting are among the cognitive domains most reliably shown to be impaired in OCD (Cohen and Ivry 1996; Kim et al. 2002; Chamberlain et al. 2006), it is conceivable that parietal lobe dysfunction, particularly within the angular and the supramarginal gyri, could contribute to the cognitive deficits evident in OCD (Menzies et al. 2008a).

Additionally, we also found evidence of microstructural-diffusivity alterations in the right medium/inferior temporal gyri. Several functional neuroimaging studies implicate the temporal lobe in OCD pathophysiology by demonstrating a significant correlation between increased glucose metabolism in this region and OCD symptomatology (McGuire et al. 1994) or consistent activation in temporal areas in response to several OCD-significant and -specific stimuli (Szeszko et al. 1999; Adler et al. 2000; Phillips et al. 2000). Indeed, both parietal and temporal regions have been implicated in the extended neuroanatomical model of OCD, predominantly because these two areas are functionally connected to the corticosubcortical OCD circuitry (Piras et al. 2013a,b2013b). Furthermore, studies investigating OCD-related alterations of WM tracts found decreased WM integrity in temporo-parietal-occipital regions and in long-range and corticocortical bundles connecting the frontal, parietal, temporal, and occipital lobes. Interestingly, structural anatomical abnormalities in these tracts positively correlate with both symptom severity and neuropsychological performance (Garibotto et al. 2010) suggesting that symptom expression, and the specific profile of cognitive impairment in OCD patients might be subtended by intrahemispheric disconnection.

In line with such findings, we found evidence of decreased integrity in the frontal portion of the SLF, a major associative tract connecting large parts of the frontal association cortices to parietotemporal association areas (Derjerine 1901). WM abnormalities were also detected in the body of CC, a structure including interhemispheric fibres connecting associative areas in parietal lobes of both hemispheres. Such decrease in intra and interhemispheric long-range connectivity might lead to functional network abnormalities within the frontotemporal and frontoparietal systems central to motor and cognitive control (Menzies et al. 2007), thus contributing to determine the symptoms and cognitive deficits associated with OCD (Zhang et al. 2011).

In sum, the reported evidence suggests that in addition to abnormalities in the classical orbitofronto-striatal loop, dysfunctions and impaired anatomical connectivity of large-scale neural systems, including lateral frontal, parietal and temporal areas, may be pathogenic for OCD.

However, our study has several limitations. First, the small sample size may limit generalization of findings, as from the initial 33 patients approached, only 20 satisfied the inclusion criteria. Nevertheless, by admitting patients with no dementia or psychiatric comorbidities, we excluded the confounding factors of neurological or other DSM-IV disorders, which would likely have additive effects on brain structure beyond those of OCD itself. Moreover, as performance deficits on tests of cognitive functioning are associated with comorbid conditions (Aycicegi et al. 2003), as for example, the relative impact of depression on executive function impairments (Basso et al. 2001), the inclusion of pure OCD allowed us to profile the exact pattern of impairment characterizing the disorder. Future studies should target to wider samples in order to increase results generalizability. Secondly, albeit symptoms of OCD can be extremely heterogeneous (Mataix-Cols et al. 2005) with, sometimes, nonoverlapping symptom patterns across patients, we grouped together patients with mixed OCD phenomenology, which may have biased study outcomes. However, the picture of widespread brain changes and altered intra and interhemispheric connectivity emerging from this study may well be accounted for by the clinical heterogeneity of OCD (Piras et al. 2013a) and considered representative of neural abnormalities underlying OCD symptoms. Although pure subtypes of OCD patients are rare and the recruitment of sufficient sample sizes of each subtype is difficult, future studies should focus on investigation of more homogeneous OCD subtypes and associated macro and microstructural specific abnormalities. Finally, while some patients in our sample were on stable doses of antipsychotic drugs (which were not included in the analyses as covariates of no interest), the high interindividual variability with respect to treatment histories, procedures, and responses should be considered as a general limitation in this kind of research. Specifically, several studies demonstrated a significant improvement in cognitive performance secondary to dopaminergic effects of selective serotonin reuptake inhibitors (Borkowska et al. 2002), while anxiolytic effects of benzodiazepines might also contribute to an improved performance in highly anxious patients (Desai et al. 1983). The fact that we observed in our medicated sample persistent neuropsychological deficits despite symptom control, would suggest that such impairments are stable trait-like features of OCD (Bannon et al. 2006). On the other hand, few available pieces of evidence confirm that psychotropic drugs can affect WM microstructure (Yoo et al. 2007), and the FA changes observed in our OCD patients might not necessarily be an index of WM pathology, but could reflect a yet unexplored part of the mechanism of action of drugs used in psychiatric treatment, or be a marker of the biological effect of psychotropic drugs on the brain (Benedetti et al. 2013). Nevertheless, this perspective is highly speculative because existing animal models have well-correlated DTI measures with WM lesions, and future studies examining WM integrity before and after treatment will contribute to clarify this issue.

## Conflict of Interest

None declared.
